# Sociodemographic and clinical characteristics of the first cohort of COVID-19 recoveries at two national treatment centres in Accra, Ghana

**DOI:** 10.4314/gmj.v54i4s.4

**Published:** 2020-12

**Authors:** Christian Owoo, Joseph A Oliver-Commey, Benedict N L Calys-Tagoe, Ebenezer Oduro-Mensah, Lawrence Ofori-Boadu, Evelyn Adjei-Mensah, Ernest Kenu, Ali Samba, Alfred E Yawson, Margaret Lartey, COVID-19 Collaborators

**Affiliations:** 1 National COVID-19 Treatment Centre, University of Ghana Medical Centre (UGMC), Accra; 2 Department of Anaesthesia, University of Ghana Medical School, College of Health Sciences, University of Ghana, Accra; 3 National COVID-19 Treatment Centre, Ga East Municipal Hospital, Ghana Health Service (GHS); 4 LEKMA Hospital, Ghana Health Service; 5 Department of Community Health, University of Ghana Medical School (UGMS), College of Health Sciences, University of Ghana, Accra, Ghana; 6 Department of Medicine and Therapeutics, University of Ghana Medical School, College of Health Sciences, University of Ghana, Accra; 7 National COVID-19 Case Management Team, Ghana; 8 Institutional Care Division, Ghana Health Service; 9 Department of Community Health, Korle-Bu Teaching Hospital, Accra, Ghana; 10 Department of Epidemiology and Disease Control, School of Public Health, University of Ghana; 11 Department of Obstetrics & Gynaecology Department, Korle-Bu Teaching Hospital, Accra, Ghana

**Keywords:** COVID-19, Ghana, Clinical characteristics, Recovery, Coronavirus

## Abstract

**Introduction:**

COVID-19 is a new disease, knowledge on the mode of transmission and clinical features are still evolving, new tests are being developed with inherent challenges regarding interpretation of tests results. There is generally, a gap in knowledge on the virus globally as the pandemic evolves and in Ghana, there is dearth of information and documentation on the clinical characteristics of the virus. With these in mind, we set out to profile the initial cohort of COVID-19 patients who recovered in Ghana.

**Methods:**

We reviewed clinical records of all confirmed cases of COVID-19 who had recovered from the two main treatment centres in Accra, Ghana. Descriptive data analysis was employed and presented in simple and relational tables. Independent t-test and ANOVA were used to determine differences in the mean age of the sexes and the number of days taken for the first and second retesting to be done per selected patient characteristics.

**Results:**

Of the 146 records reviewed, 54% were male; mean age of patients was 41.9 ± 17.5 years, nearly half were asymptomatic, with 9% being severely ill. The commonest presenting symptoms were cough (22.6%), headache (13%) and sore throat (11%) while the commonest co-morbidities were hypertension (25.3%), diabetes mellitus (14%) and heart disease (3.4%).

**Conclusion:**

COVID-19 affected more males than females; nearly half of those infected were asymptomatic. Cough, headache and sore throat were the commonest symptoms and mean duration from case confirmation to full recovery was 19 days. Further research is required as pandemic evolves

**Funding:**

None declared

## Introduction

Coronavirus belongs to a large family of viruses that cause a wide range of illnesses ranging from mild to severe. In the past two decades, coronaviruses have caused 2 major pandemics i.e. severe acute respiratory syndrome (SARS) and Middle East respiratory syndrome (MERS).^1^ Respiratory droplets and direct contacts have been identified as the main route of transmission. Fever and cough are the main symptoms reported^2^

Following the outbreak of the novel Coronavirus disease (COVID-19) in December 2019, there has been an unprecedented geographical spread to more than 210 countries and territories and 2 international conveyances^3^ around the world. This novel coronavirus disease (COVID-19) is a new virus linked to the same family of viruses as SARS. The virus is transmitted through direct contact with respiratory droplets from an infected person or touching contaminated surfaces.^4^

The SARS-COV-2 (the virus that causes COVID-19) is a β-coronavirus which is an enveloped non-segmented positive-sense RNA virus, and it has the unique ability to infect mammals.^5^ The Angiotensin converting enzyme (ACE2) receptor, which can be found in the lower respiratory tract of humans, is a known cell receptor for coronaviruses and regulates both the cross-species and human-to-human transmission. The virus cell interaction produces a diverse set of immune mediators against the invading virus. It causes an inflammatory response in the lower airway which leads to lung injury. The viral particles invade the respiratory mucosa firstly and infect other cells, triggering a series of immune responses and production of cytokine storm in the body which is associated with the critical condition of infected patients.^6^ COVID-19 spreads to the respiratory tract by droplets, respiratory secretions and direct contact for low infective dose. SARS-COV-2 has also been isolated from fecal swabs of severe pneumonia patients. The incubation period is 1–14 days with an average of mostly 3–7 days. C0VID-19 is contagious during the latency period. In a Chinese study, the median age of victims was found to be between 47–59 years.^7^

As at the end of April 2020, there were 3.18 million recorded cases of Covid-19 worldwide with 224,172 deaths. During the same period, Africa had recorded 26,663 cases with 973 deaths.^8^ Ghana, recorded its first 2 cases of Covid-19 on the 12^th^ of March 2020 and by the end of April 2020, the number of cases has increased to 2074, with 188 recoveries and 17 deaths.^9^

COVID-19 is a new disease, knowledge on the mode of transmission and clinical features are still evolving, new tests are being developed with inherent challenges regarding interpretation of tests results. In addition, treatment regimens are generally in the pilot stages and data to support effective treatment regimens still debatable. There is generally, a gap in knowledge on the virus globally as the pandemic evolves and in Ghana, there is dearth of information and documentation on the clinical characteristics of the virus. With all these in mind, we set out to profile the initial cohort of COVID-19 patients who recovered in Ghana. This we believe will provide some answers to the many unanswered questions surrounding COVID-19 within the Ghanaian context.

## Methods

### Study design

This study involved a review of the clinical records of all confirmed cases of covid-19 who had received treatment and recovered from the two main treatment centers in Accra, the epicenter of Ghana, namely; The Ga East Municipal hospital (GEMH) and the University of Ghana Medical center (UGMC). The GEMH, which is managed by the Ghana Health Service (GHS), is the largest covid-19 treatment center within the country and has managed over 180 cases as at the end of April, 2020. The UGMC, on the other hand, is jointly managed by the Ministry of Health (MoH) and the University of Ghana (UG) and has managed over 25 cases of covid-19 within the same period.

### Study population

We reviewed the records of the first cohort of confirmed COVID-19 cases who had recovered at the GEMH and UGMC. A confirmed case of COVID-19 was defined as a person with laboratory confirmation of COVID-19 using real-time reverse-transcriptase-polymerase-chainreaction (RT-PCR) assay of nasal and/or pharyngeal swab specimens irrespective of clinical signs and symptoms.^10^ Recovery from COVID-19 was defined as a confirmed COVID-19 patient who subsequently tests negative on two consecutive RT-PCR tests (with samples taken at least 24 hours apart) and is clinically asymptomatic.^11^

### Data collection

An abstraction form was used to abstract the relevant variables from the clinical records of the patients who have recovered from COVID-19. Altogether, 146 records were included in the analysis presented in this study. All cases with missing socio-demographic data were excluded from the analysis.

### Variables

Variables considered in this analysis were demographics (age, sex, educational level, nationality, country of permanent residence), exposure history, symptoms and co-morbid conditions, type of sample taken, disease severity and treatment modalities. Based on Strengthening the Reporting of Observational Studies in Epidemiology (STROBE) recommendation for cross-sectional study design, missing responses were strictly excluded in our analysis^12^

### Data analysis

Descriptive data analysis was carried out and presented in frequencies and percentages on all variables considered in the study. An independent two-sample t-test was used to determine any differences in the mean age for males and females and the mean number of days taken for the first and second retesting to be done at the two treatment centres. ANOVA was used to determine the mean differences in mean duration for recovery between the various categories of disease severity and case classification. Stata 16 was used for all analysis.

### Patient and public involvement

This study did not directly involve the use of patients and the public. No patients were involved in the design of the study neither were any patients recruited during the study. However, the medical records of patients were reviewed. Since no patients were recruited, the results will not be disseminated to individual patients but rather to the Ghana Health service and by extension the entire Ministry of Health to inform management of COVID-19 in Ghana

### Ethics approval and consent to participate

Ethical clearance was obtained from GHS Ethics Review Committee (GHS-ERC 006/05/20). Permissions and letters of support were obtained from the heads of the institutions (GEMH and UGMC) where the data was abstracted. Additionally, codes, rather than personal identifiers were used throughout the process of data abstraction and analysis to ensure anonymity and maintain patient confidentiality.

## Results

In total, the records of 146 cases of COVID-19 were reviewed and included in this analysis. Most of the patients (90%) were managed at the Ga East Municipal hospital. Of the 146 patients, 54% were male. The overall mean age of the patients was 41.9 ± 17.5 years [male vrs female = 41.6 ±17.4 vrs 42.3 ±17.8; p-value = 0.825]. Fifty four percent (54%) were between the ages of 20–49 years with 6% being 70 years or older.

Also, 84% were permanent residents in Ghana and 59% had a history of international travel within the 14 days prior to being diagnosed with COVID-19 and were thus classified as imported cases. Throat swabs were used for diagnosis for the majority (70%) of them followed by sputum (19%). Table 1 highlights the characteristics of these individuals.

**Table 1:** Descriptive characteristics of initial cohort of fully recovered COVID-19 patients

Demographic characteristics	N (%)
**Sex**	
**Female**	67(45.9)
**Male**	79 (54.1)
**Age group**	
**<19**	13 (8.9)
**20–29**	37 (25.3)
**30–39**	21 (14.4)
**40–49**	21 (14.4)
**50–59**	26 (17.8)
**60–69**	19 (13.0)
**70+**	9 (6.2)
**Ghana**	122 (83.6)
**Other**	3 (2.1)
**UAE**	2 (1.4)
**UK**	17 (11.6)
**Nigeria**	2 (1.4)
**No formal education**	8 (5.5)
**Primary**	23 (15.7)
**Secondary**	37 (25.4)
**Tertiary**	78 (53.4)
**No**	141 (96.6)
**Yes**	5 (3.4)
**No**	59 (40.7)
**Yes**	86 (59.3)
**No**	128 (88.9)
**Yes**	16 (11.1)
**No**	106 (73.6)
**Yes**	16 (11.1)
**Unknown**	22 (15.3)
**Airplane**	10 (7.6)
**Home**	31 (23.7)
**Market**	3 (2.3)
**Social gathering**	2 (1.5)
**Workplace**	13 (9.9)
**Unknown**	72 (55.0)
**Type of sample**	
**Nasal swab**	12 (8.2)
**Nasopharyngeal** **swab**	4 (2.7)
**Sputum**	28 (19.2)
**Throat swab**	102 (69.9)
**Ga East**	132 (90.4)
**UGMC**	14 (9.6)
**Nationality**	
**Dual**	6 (4.1)
**Ghanaian**	133 (91.1)
**Non-Ghanaian**	7 (4.8)

**Figure 1:**
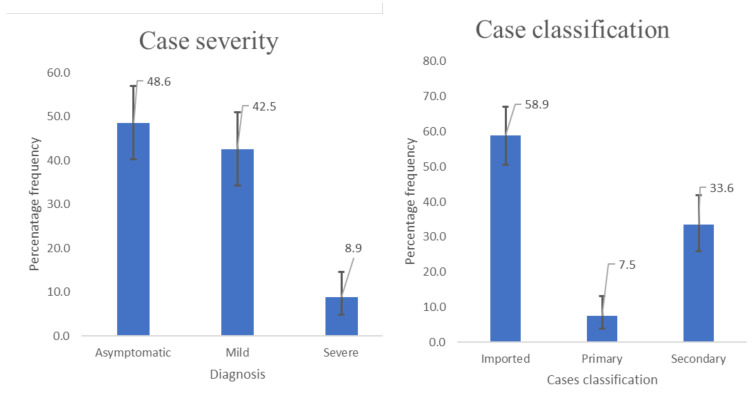
Case severity and classification of recovered COVID-19 cases

As shown in Figure 1, nearly half (49%) of these individuals were asymptomatic at the time of diagnosis, with 9% being severely ill. The most common presenting symptoms were cough (22.6%), headache (13%) and sore throat (11%). Other presenting symptoms are as indicated in Table 2. Of the 146 patients 33.6% had one or more coexisting medical conditions.

**Table 2:** Presenting symptoms among the cohort of fully recovered COVID-19 patients in Ghana (n=146)

Patient symptoms	Yes
	n(%)
**Symptoms and signs**	
**Cough**	33(22.6)
**Headache**	19(13.0)
**Sore throat**	16(11.0)
**Myalgia**	13(8.9)
**Anorexia**	11(7.5)
**Fever (≥38 °C) or history of fever**	10(6.8)
**Fatigue**	9(6.2)
**Runny nose**	8(5.5)
**Shortness of breath**	7(4.8)
**Diarrhoea**	7(4.8)
**Chills**	6(4.1)
**Arthralgia**	6(4.1)
**Nausea**	3(2.1)
**Vomiting**	1(0.7)
**Rash**	1(0.7)

The most common coexisting conditions were hypertension (25.3%), diabetes mellitus (14%), heart disease (3.4%) and asthma-requiring medication (2.7%). Gastrointestinal symptoms such as nausea (0.7%) and vomiting (0.7%) were among the least reported symptoms. Information on alcohol intake and tobacco smoking was available in 132 of the 146 records reviewed. Of these, more than a quarter, (28%) had a history of alcohol intake and 6% had a history of smoking tobacco. Five out of the 146 (3.4%) developed acute respiratory distress syndrome (ARDS)^13^ while 3 (2.1%) had pneumonia (as evidenced by chest x-ray/chest CT-scan) as complications (Table 3).

**Table 3:** Co-morbidities/Complications among the cohort of fully recovered COVID-19 patients (n=146)

Co-morbidities/Complications	Yes
	n (%)
**Complications encountered**	
**Acute respiratory distress syndrome** **(ARDS)**	5(3.4)
**Pneumonia by chest X-ray**	3(2.1)
**Pre-existing morbidity**	
**Cancer**	3(2.1)
**Diabetes**	20(13.7)
**Hypertension**	37(25.3)
**HIV/other immune deficiency**	1(0.7)
**Heart disease**	5(3.4)
**Asthma (requiring medication)**	4(2.7)
**Chronic kidney disease**	2(1.4)
**History of smoking tobacco**	8(6.1) *
**History of alcohol intake**	37(28.0) *

* information on these variables was available in 132 of the 146 records reviewed

The mean duration for a confirmed case of COVID-19 to have an initial negative test result was 13.4 days and 19 days for the second consecutive negative test, both of which were required before a patient can be said to have recovered from COVID-19.

The variations in this duration with respect to selected characteristics are shown in Table 4. There was a significant difference in this duration between patients managed at the two facilities from which patients' records were reviewed.

**Table 4:** Mean number of days taken to conduct repeat tests among fully recovered COVID-19 patients per selected characteristics

Demographic characteristics	Days for 1st negative	Days for 2nd negative	2nd and 1st days difference
	µ[95%CI]	µ[95%CI]	µ[95%CI]
**Overall**	13.4[12.4–14.4]	19.0[18.0–20.0]	5.6[5.2–5.9]
**Case severity**			
**Asymptomatic**	12.7[11.5–13.9]	18.3[17.2–19.5]	5.6[5.1–6.2]
**Mild**	14.2–12.5–16.0]	19.6[17.9–21.3]	5.3[4.7–5.8]
**Severe**	13.6[9.8–17.4]	20.7[17.2–24.2]	7.1[5.2–9.0]
**Case classification**	**	**	***
**Imported**	12.1[11.3–13.1]	18.2[17.2–19.2]	6.0[5.5–6.5]
**Primary**	12.8[9.8–14.7]	17.2[14.4–20.0]	4.4[3.3–5.5]
**Secondary**	16.4[13.9–18.9]	21.3[19.1–23.6]	4.9[4.3–5.6]
**Treatment centre**	***	***	
**Ga East**	14.1[13.1–15.2]	19.7[18.7–20.7]	5.6[5.1–5.9]
**UGMC**	7.4[6.6–8.3]	13.1[12.8–13.4]	5.7[5.1–6.3]

NOTE: P-value notation: **=p-value<0.01; ***p-value<0.001

The UGMC had a much shorter mean duration of 7.4 days and 13.1days compared with 14.1 days and 19.7 days for those managed at GEMH for the first and second consecutive negative tests respectively. There was however no difference in the mean duration (5.6 days) between the first and second consecutive negative tests at both centres. There was also no observed difference in the mean duration based on case severity.

## Discussion

As part of efforts to describe the COVID-19 pandemic in the Ghanaian setting, we reviewed the medical records of the first cohort of COVID-19 patients to have recovered at the Ga East Municipal hospital and the University of Ghana Medical Center. This report, to the best of our knowledge, is the first documented profiling of COVID-19 cases in Ghana. A total of 146 patient records were reviewed.

The mean age of patients in this study was 41.9 ± 17.5 years. This is much lower than the values of 49 and 50 years reported from studies in Wuhan^2^, ^5^ where the pandemic started. It is also lower than what was reported in Russia (46 years)^14^ and by the W.H.O. (51 years).^15^ This observed difference is most likely due to the difference in population structure of the study settings; Ghana has a younger population compared to China and Russia. Another plausible reason for the lower mean age of patients in this study is the source of the cohort of patients. Majority (86 out of 146) of them were cases that were picked up during the mandatory quarantine and isolation of travelers arriving in Ghana after the Executive decision to impose a 14 day-mandatory quarantine on all travelers arriving in Ghana following the closure of the country's international borders to human transport. The mean age of active travelers would be expected to be lower than that of the general population and this may have impacted on the age distribution of this initial cohort of COVID-19 patients in Ghana. Surprisingly, another study in Wuhan^16^ reported a median age of 34 years. This study however, involved only 13 participants of which two were children aged 2 and 15 years and this explains the relatively lower median age. As reported by other studies^2^,^5^,^16^, majority of the patients (54%) were males. This male preponderance is not fully understood and may require further investigation.

Contrary to reports from other studies^2^, ^17^ in which majority of patients presented with fever (>90%) and cough (59–76%), nearly half (49%) of the patients in this study were asymptomatic at the time of diagnosis with only 6.8% having fever or reporting a history of fever and less than a quarter (23%) having cough at the time of diagnosis. This significantly lower proportion of patients with symptoms at the time of diagnosis could be the result of the approach adopted by Ghana in identifying cases of COVID-19. After the first few imported cases of COVID-19 in Ghana and the institution of mandatory quarantine of all travelers arriving into Ghana, all the quarantined travelers were mandatorily tested for the SARS-COV-2 virus irrespective of clinical presentation. It is plausible to expect that a proportion of this cohort may have contracted the disease while in transit through major travel hubs and international airports in Asia, Europe and Northern America which by then were major global COVID-19 epicentres. Therefore, though these individuals tested positive, they were likely to be presenting much earlier in their disease process.

In addition to the routine surveillance, the country also adopted an enhanced surveillance approach^9^ where all contacts of cases were identified and tested. Community members of these cases (within a 2km radius) were also identified and tested whether they had symptoms or not. This made it possible for individuals to be diagnosed and isolated before the onset of symptoms in a lot of cases. This has the potential of reducing the number of contacts made by these individuals and thus reducing the rate of spread of the virus.

Almost a third (33.6%) of patients in this study had an underlying medical condition. Huang et al^5^, reported similar findings (32%), however, Wang et al^2^ reported much higher values (46.4%). About a quarter (25.3%) had hypertension and 13.7% had diabetes mellitus as coexisting medical conditions. These figures closely reflect the prevalence of these conditions in the general Ghanaian population.^18^, ^19^ This prevalence of hypertension among the patients was higher than that reported by Huang et al^5^ (15%) but lower than what was found by Wang et al^2^ (31.2%). Again, the prevalence of diabetes mellitus (13.7%) was higher than that reported by Wang et al^2^ (10.1%) but lower than what was found by Huang et al^5^ (20%). These differences could be the result of sample size and sampling differences.

Contrary to findings from other settings that reported ICU admission rate of between 26 and 32%^2^, ^5^, only 5.5% (8/146) of our patients were admitted to the ICU (3 had severe pneumonia while 5 had acute respiratory distress syndrome). The relatively low ICU admission rate cannot readily be explained by this study however, it may be due to a combination of factors including the relatively younger age of our patients as well as the early diagnosis due to the enhanced surveillance adopted by Ghana. There have also been speculations about role of the viral variants that are causing disease in Sub-Saharan Africa including Ghana and their interaction with the genetic makeup of our population. We, however, did not assess that in this study.

In Ghana, recovery from COVID-19 is defined as “a previously confirmed case subsequently having two consecutive negative tests for which samples were taken at least 24 hours apart”. Based on this definition, the overall mean durations for patients to have their initial and second negative tests (to be declared fully recovered) were 13.4 days and 19 days respectively. This was much longer than the 12 days reported by a study in Singapore^20^ for full recovery. This observed difference could be attributed to the different testing strategies used in the two settings. In the Singapore study^20^, confirmed cases of COVID-19 were tested daily and so it was easier to tell exactly the day on which a case first becomes negative. In Ghana however, there were some challenges with sampling and testing earlier in the epidemic in Ghana which made daily testing of confirmed cases impossible. These challenges include the limited testing capacity of our laboratories at the start of the epidemic which resulted in a backlog of samples leading to delay in obtaining test results. The other challenge was a policy directive to test confirmed cases after 10–14 days rather than daily or weekly because most of the cases that had the initial weekly testing still had positive results after the first week. This, coupled with the initial limited laboratory capacity, informed that policy directive and may be accounting for the difference in the overall mean duration for recovery.

It is however worth noting, that there was a significant difference in the mean recovery time between the two centres even though they followed the same treatment protocol to a large extent; UGMC had a mean duration of 7.4 days and 13 days as against 14 days and 20 days for GEMH for the initial and consecutive negative tests. Most of the initial confirmed cases were managed at GEMH at a time when there was limited laboratory testing capacity with all its attendant spill overs. Subsequent confirmed cases were managed at UGMC at a time when there had been significant improvement in laboratory capacity and the backlog of samples had all been cleared, thus testing could be done, and results obtained within a shorter period.

## Conclusion

In this initial profiling of COVID-19 cases in Ghana, males were found to be more infected than females; nearly half of those infected were asymptomatic with the commonest symptoms being cough, headache and sore throat. The overall mean duration from case confirmation to full recovery was19 days even though there was a significant difference in this mean duration between the two centres. Further studies might be required to determine more precisely how long it takes for a confirmed case of COVID-19 to fully recover in Ghana.

## References

[1] Zhou P, Yang X, Wang X, Hu B, Zhang L, Zhang W (2020). A pneumonia outbreak associated with a new coronavirus of
probable bat origin. Nature.

[2] Wang D, Hu B, Hu C, Zhu F, Liu X, Zhang J (2020). Clinical Characteristics of 138 Hospitalized Patients With 2019
Novel Coronavirus-Infected Pneumonia in Wuhan, China. JAMA.

[3] Worldometer (2020). COVID-19 Coronavirus Pandemic.

[4] Sohrabi C, Alsafi Z, O'Neill N, Khan M, Kerwan A, Al-Jabir A (2020). World Health Organization declares global emergency: A review of
the 2019 novel coronavirus (COVID-19). International Journal of Surgery.

[5] Huang C, Wang Y, Li X, Ren L, Zhao J, Hu Y (2020). Clinical features of patients infected with 2019 novel
coronavirus in Wuhan, China. Lancet.

[6] Jia HP, Look DC, Shi L, Hickey M, Pewe L, Netland J (2005). ACE2 Receptor Expression and Severe Acute Respiratory Syndrome
Coronavirus Infection Depend on Differentiation of Human Airway
Epithelia. Journal of Virology.

[7] Jin YH, Cai L, Cheng ZS, Cheng H, Deng T, Fan YP (2020). A rapid advice guideline for the diagnosis and treatment of 2019
novel coronavirus (2019-nCoV) infected pneumonia (standard
version). Military Medical Research.

[8] World Health Organization situation report on COVID-19 No. 102.

[9] COVID-19 Updates | Ghana.

[10] Guan W, Ni Z, Hu Y, Liang W, Ou C, He J (2020). Clinical Characteristics of Coronavirus Disease 2019 in
China. N Engl J Med [Internet].

[11] World Health Organization Clinical management of COVID-19, interim guidelines (May 20220).

[12] von Elm E, Altman DG, Egger M (2007). The Strengthening the Reporting of Observational Studies in
Epidemiology (STROBE) Statement: Guidelines for Reporting Observational
Studies. Ann Intern Med.

[13] ARDS definition.

[14] https://www.statista.com/statistics/1111350/russiacovid-19-patients-average-age-by-gender/.

[15] https://www.who.int/docs/default-source/coronaviruse/situation-reports/20200418-sitrep-89-covid-19.pdf.

[16] Chang D, Lin M, Wei L (2020). Epidemiologic and Clinical Characteristics of Novel Coronavirus
Infections Involving 13 Patients Outside Wuhan, China. JAMA.

[17] Chen T, Wu D, Chen H, Yan W, Yang D, Chen G (2020). Clinical characteristics of 113 deceased patients with
coronavirus disease 2019: retrospective study. BMJ.

[18] Baffour Awuah R, De-Graft Aikins A, Dodoo FNA, Meeks KAC, Beune EJAJ, Klipstein-Grobusch K, Addo J, Smeeth L, Bahendeka SK, Agyemang C (2019). Psychosocial factors and hypertension prevalence among Ghanaians
in Ghana and Ghanaian migrants in Europe: The RODAM study. Health Psychology Open.

[19] Asamoah-Boaheng M, Sarfo-Kantanka O, Boaheng Tuffour A, Eghan B, Mbanya JC (2019). Prevalence and risk factors for diabetes mellitus among adults in
Ghana: a systematic review and meta-analysis. Int Health.

[20] Young BE, Ong SWX, Kalimuddin S, Low JG, Tan SY, Loh J (2020). Epidemiologic Features and Clinical Course of Patients Infected
with SARS-CoV-2 in Singapore. J Am Med Assoc.

